# Supplementary material for: Microsatellite frequencies vary with body mass and body temperature in mammals, suggesting correlated variation in mutation rate
Source: PeerJ. 2014 Nov 6;2:e663. doi: 10.7717/peerj.663 (PMC4226647; doi:10.7717/peerj.663)
Supplement: Figure S1 — The program takes one character at a time sequentially from the genome file. It is assumed that the overwhelming majority of characters are the bases A, C, G, T and N in either upper or lower case. All characters with codes below 65 (=‘A’) or above 122 (‘z’) are ignored. The script can be used by pasting into a C++ compiler and editing the path where the genome files are located. [file peerj-02-663-s004.pdf]

```
#include <iostream.h>
#include <stdlib.h>
#include <fstream.h>
#include <string.h>
#include <stdio.h>
#include <math.h>

int main ()
{
  long micros[51][50];  // array to contain microsatellite counts

  // get input file name
  cout << "name of input file without entension ";
  char species[10];
  cin >> species;

  // initialise default parameter values
  int computer=2;
  int startrpt=1;
  int endrpt=4;
  int purity=0;
  int fay=1;
  int look=0;
  char change='x';

  // create an editable option table with options for repeat type, path for two computers, whether to look
  // for interrupted repeats or not, whether input genome file has '.fa' extension, or a look-see version
  while (change!='y') {
  cout << "repeats are 1=AC, 2=AG, 3=AT, 4=CG\n\n";
  cout << "computer: 1 = old, 2 = new . . . . . " << computer << "  <a>\n";
  cout << "interrupts: 0=pure, 1=interrupted  . " << purity << "  <b>\n";
  cout << "start repeat number        . . . . . " << startrpt << "  <c>\n";
  cout << "end repeat number          . . . . . " << endrpt << "  <d>\n";
  cout << "add <fa> extension (0=no, 1=yes) . . " << fay << "  <e>\n";
  cout << "just look at file contents (1=yes) . " << look << "  <f>\n\n";
  cout << "continue= <y> change= letter of parameter   ";
  cin >> change;
  cout << "\n\n";
  switch(change) {
      case 'a':
           computer=3-computer;
           break;
      case 'b':
           purity=1-purity;
           break;
      case 'c':
           cout << "change start repeat to: ";
           cin >> startrpt;
           if (endrpt<startrpt) endrpt=startrpt;
           break;
      case 'd':
           cout << "change end repeat to: ";
           cin >> endrpt;
           if (endrpt<startrpt || endrpt>4) cout << "invalid option, reenter\n";
           break;
      case 'e':
           fay=1-fay;
           break;
      case 'f':
           look=1-look;
           break;
      default:
           if (change!='y') cout << "not a valid option, check case, try again \n\n";
           break;
           }
  cout << "\n\n\n";
  }

  // define variable names
  char motif[4];
  int bs1, bs2;
  char outfile[100];
```

```
// open input file and test whether open
char infile[200];
if (computer==2) strcpy(infile, "Z:\\Desktop\\Laura\\");
else strcpy(infile, "C:\\Users\\BillAmos\\Desktop\\Laura\\");
strcat(infile, species);
strcat(infile, "_genome");
if (fay==1) strcat(infile,".fa");
ifstream in(infile);
if (!in) cout << "not open for " << infile << "\n";
else cout << infile << " is open\n";

// an option just to output one line at a time to the screen
if (look==1) {
   int test=0;
   char oneline[200];
   while (test<100) {
         in.getline(oneline,200);
         cout << oneline << "\n";
         cin >> test;
         }
   }

// analyse each repeat separately, defining the component bases as ASCI codes
for (int rpt=startrpt; rpt<=endrpt; rpt++) {
   switch(rpt) {
       case 1:
            strcpy(motif,"_AC");
            bs1=65;
            bs2=67;
            break;
       case 2:
            strcpy(motif,"_AG");
            bs1=65;
            bs2=71;
            break;
       case 3:
            strcpy(motif,"_AT");
            bs1=65;
            bs2=84;
            break;
       case 4:
            strcpy(motif,"_CG");
            bs1=67;
            bs2=71;
            break;
       }

   // zero counter
   for (int i=0; i<51; i++) {
       for (int j=0; j<50; j++) micros[i][j]=0;
       }

   // create and open output file based on parameter values
   strcpy(outfile,species);
   strcat(outfile,motif);
   if (purity==1) strcat(outfile, "_irupt.txt");
   else strcat(outfile,".txt");
   ofstream out(outfile);
   if (!out) cout << "not open for " << outfile << "\n";

   // initialise and set default values for variables
   char a, b;
   long x;
   double cntall=0;
   int micro=0;
   b='x';
   int z=0;
   int b1=0;
   int b2=0;
   int b3=0;
   int z1=0;
   int z2=0;
   int first=0;
   int irupt=0;
```

```
unsigned long GC=0;
unsigned long AT=0;

// work through input file taking a line at a time
while (!in.eof()) {
   b3=0;

   // take a  character, ignoring anything that is not a letter, convert to upper case if needed
   while ((b3<65 || b3>122) && !in.eof()) b3=in.get();
   if (b3>89 && b3<123) b3-=32;

   // count AT and GC bases
   if (b3==65 || b3==84) AT++;
   if (b3==67 || b3==71) GC++;

   // if the target motif is found, reset counters and start looking for subsequent repeats
   if (b2==bs1 && b3==bs2) {
      micro=1;
      irupt=0;
      int test=0;
      while (test<(1+purity) && !in.eof()) {
          z1=0;
          z2=0;
          // take the next two characters that are letter, convert to upper case and count AT / GC
          while ((z1<65 || z1>120) && !in.eof()) z1=in.get();
          while ((z2<65 || z2>120) && !in.eof()) z2=in.get();
          if (z1>89 && z1<123) z1-=32;
          if (z2>89 && z2<123) z2-=32;
          if (z1==65 || z1==84) AT++;
          if (z1==67 || z1==71) GC++;
          if (z2==65 || z2==84) AT++;
          if (z2==67 || z2==71) GC++;
          cntall+=2;

          // if new bases match the motif, count another repeat, if not, maybe an interruption
          if (z1==bs1 && z2==bs2) micro++;
          else {
              test++; // interruption counter
              if (test==1) {
                 irupt=micro;
                 micro=0;
                 }
              }
          }

      // if minimal conditions for an acceptable microsatellite are met then count
      if ((irupt>2 || micro>2) && irupt<50 && micro<50) {
         micros[irupt][micro]++;
         if (irupt+micro+1<50) micros[50][irupt+micro+1]++;
         if (irupt==10) cout << micros[irupt][0] << "\n";  // output longer counts to screen
         }
      b1=z1;
      b2=z2;
      } // if the pair of bases does not match the repeat unit, exit the loop
   else {
      // shuffle bases back such the b1 and b2 hold the two bases prior to the next one to be read
      b1=b2;
      b2=b3;
      cntall++;
      }
   }

// at print microsatellite counts to the output file and close, return to beginning of input file
for (int j=0; j<51; j++) {
    out << j;
    for (int k=0; k<50; k++) out << " " << micros[j][k];
    out << "\n";
    }
out << "\n";
out << cntall << " " << AT << " " << GC <<"\n";
out.close(); // close output file
in.clear(); // go to beginning of input file to search for next motif
in.seekg(0,ios::beg);
}
```

```
  in.close();
  return 0;
}
```
