# Supplementary figures and images for: Microsatellite frequencies vary with body mass and body temperature in mammals, suggesting correlated variation in mutation rate
Source: PeerJ. 2014 Nov 6;2:e663. doi: 10.7717/peerj.663 (PMC4226647; doi:10.7717/peerj.663)

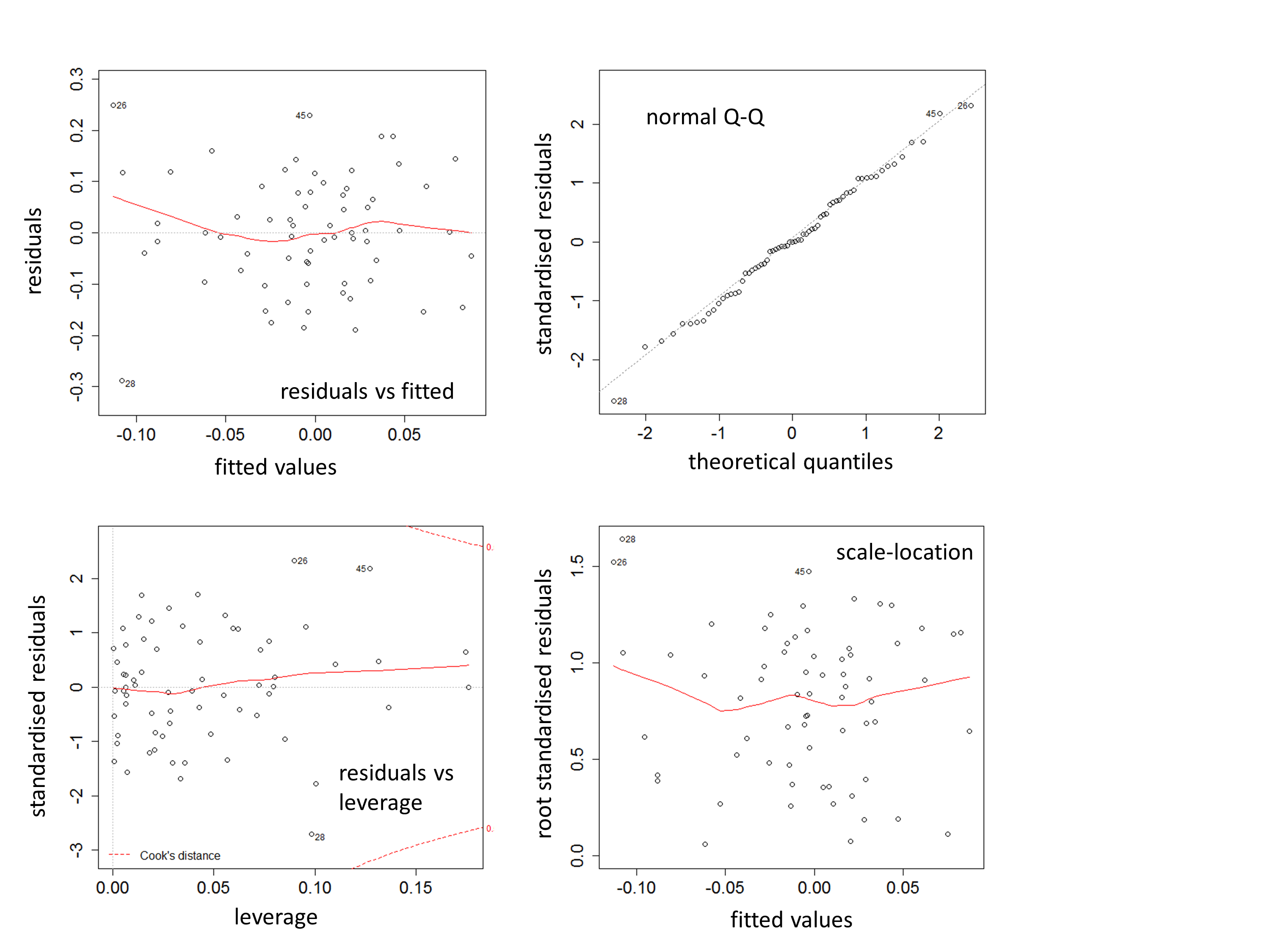

Supplement: Figure S2 — The four plots were obtain using the R command “plot(model)” and are typical for models where, in particular, the major outlier node separating primates and rodents has been removed. [file peerj-02-663-s005.png]

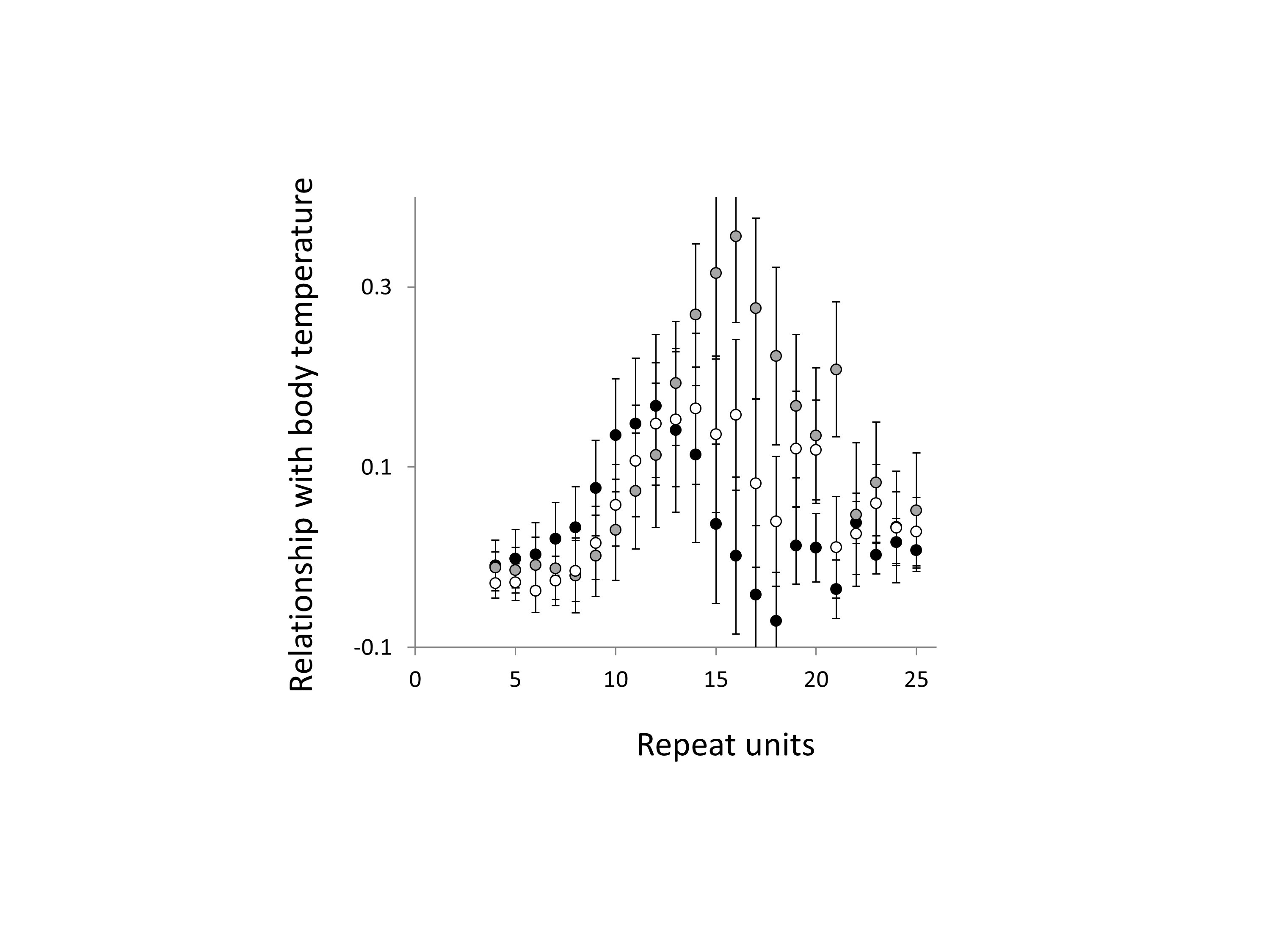

Supplement: Figure S4 — Three trinucleotide microsatellite motifs were analysed (AAC, AGG and ATT = black, grey and white data series respectively). The vertical axis is the estimate of the slope of the relationship between body temperature and loge microsatellite frequency, corrected for body mass and the genome’s GC%. All variables are included as phylogenetically independent contrasts. One major outlier was removed. Error bars are one standard error of the estimate. [file peerj-02-663-s007.png]

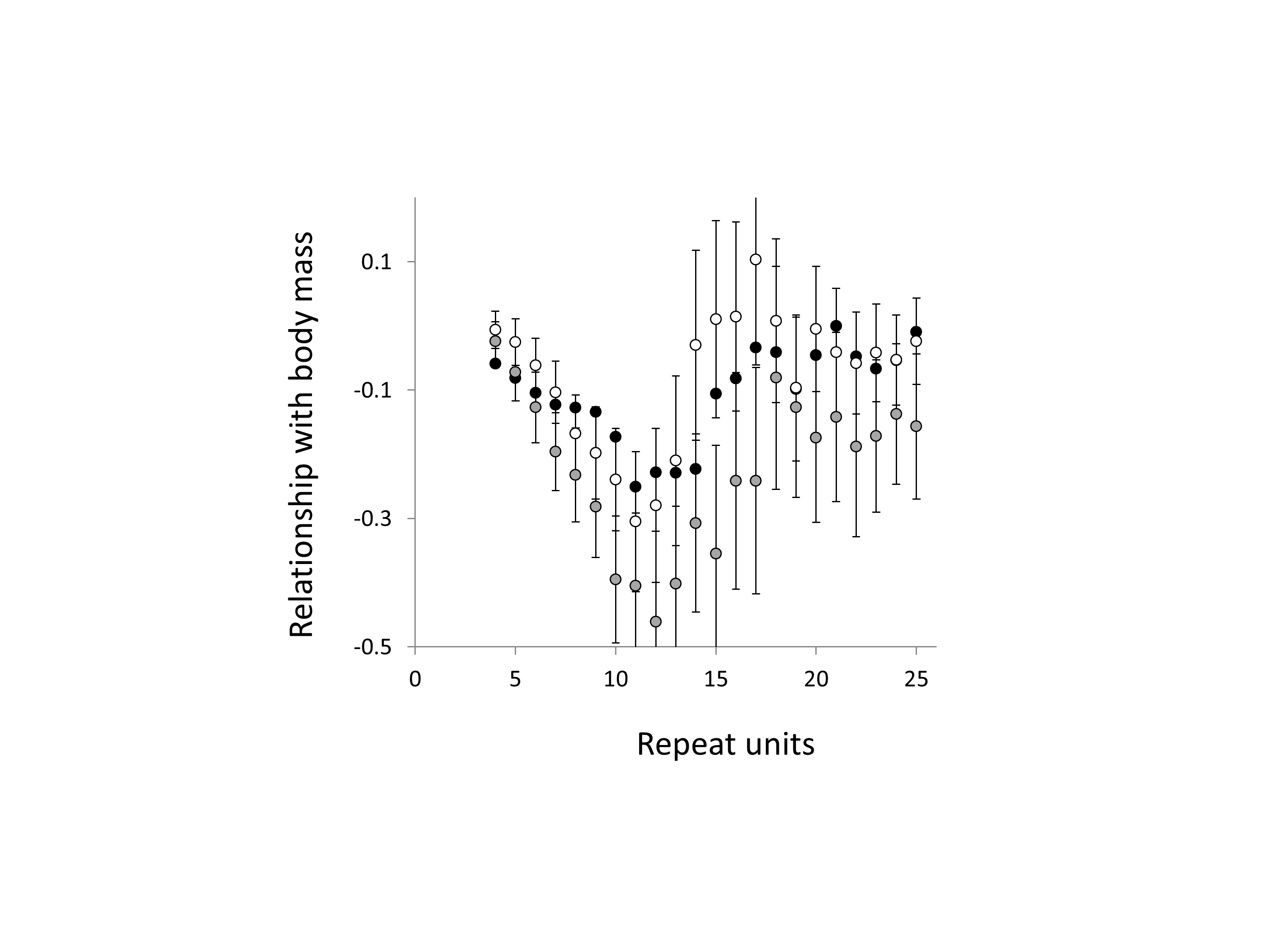

Supplement: Figure S5 — Three trinucleotide microsatellite motifs were analysed (AAC, AGG and ATT = black, grey and white data series respectively). The vertical axis is the estimate of the slope of the relationship between body mass and loge microsatellite frequency, corrected for body temperature and the genome’s GC%. All variables are included as phylogenetically independent contrasts. One major outlier was removed. Error bars are one standard error of the estimate. [file peerj-02-663-s008.png]

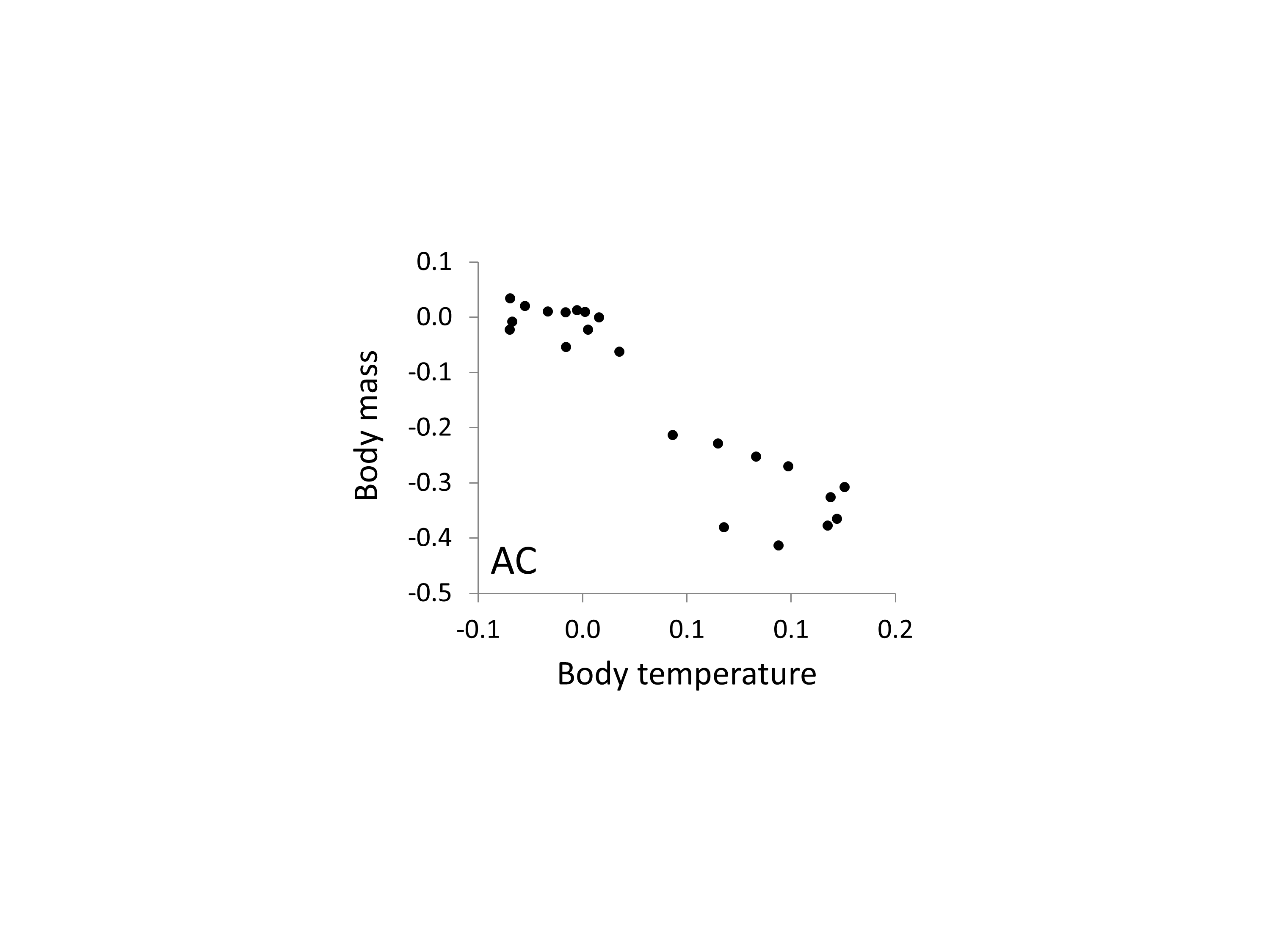

Supplement: Figure S6 — The two axes correspond to the estimates of the two phenotypes taken from Figs. 5 and 6 and show a broadly inverse relationship. [file peerj-02-663-s009.png]

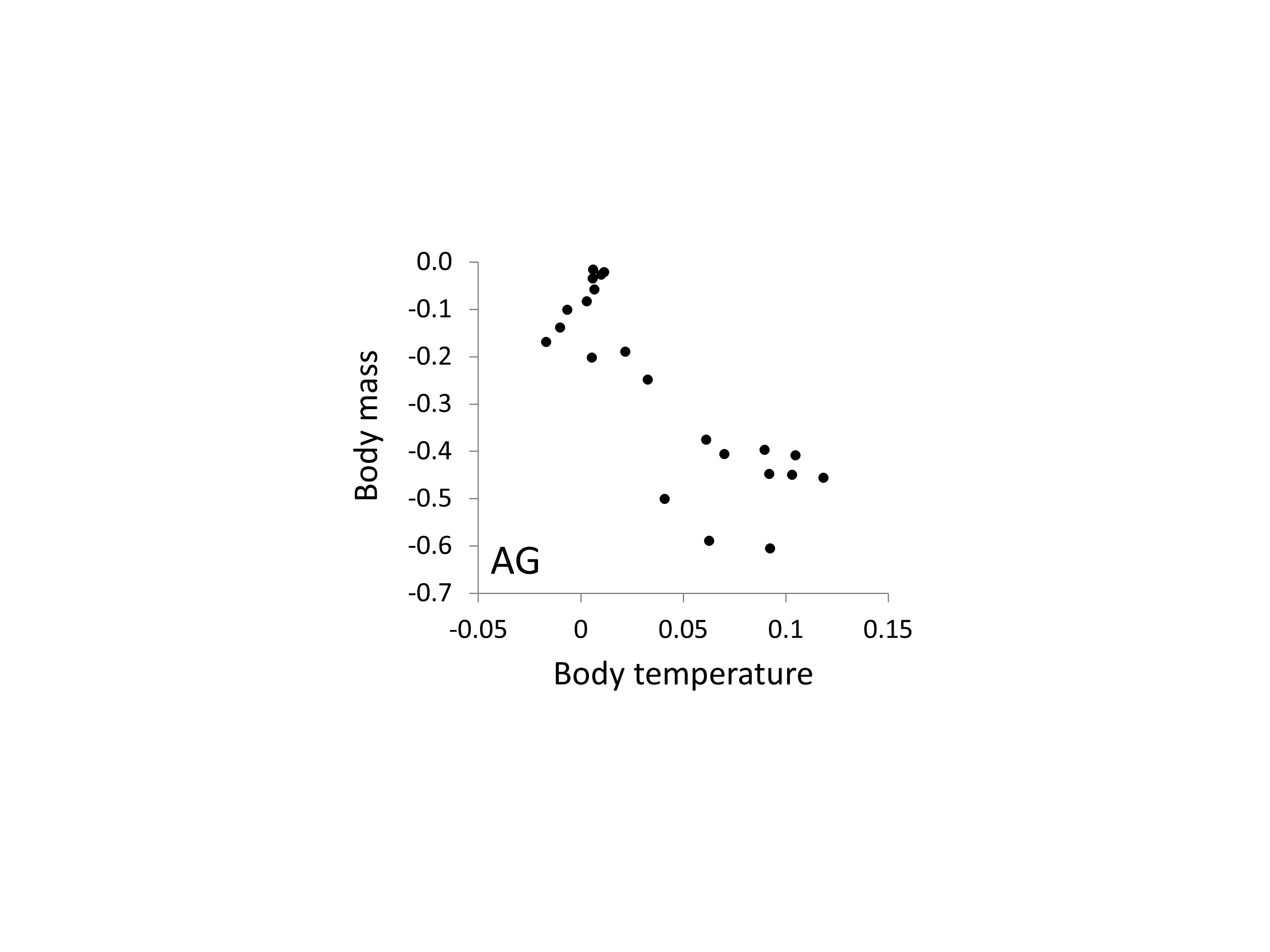

Supplement: Figure S7 — The two axes correspond to the estimates of the two phenotypes taken from Figs. 5 and 6 and show a broadly inverse relationship. [file peerj-02-663-s010.png]

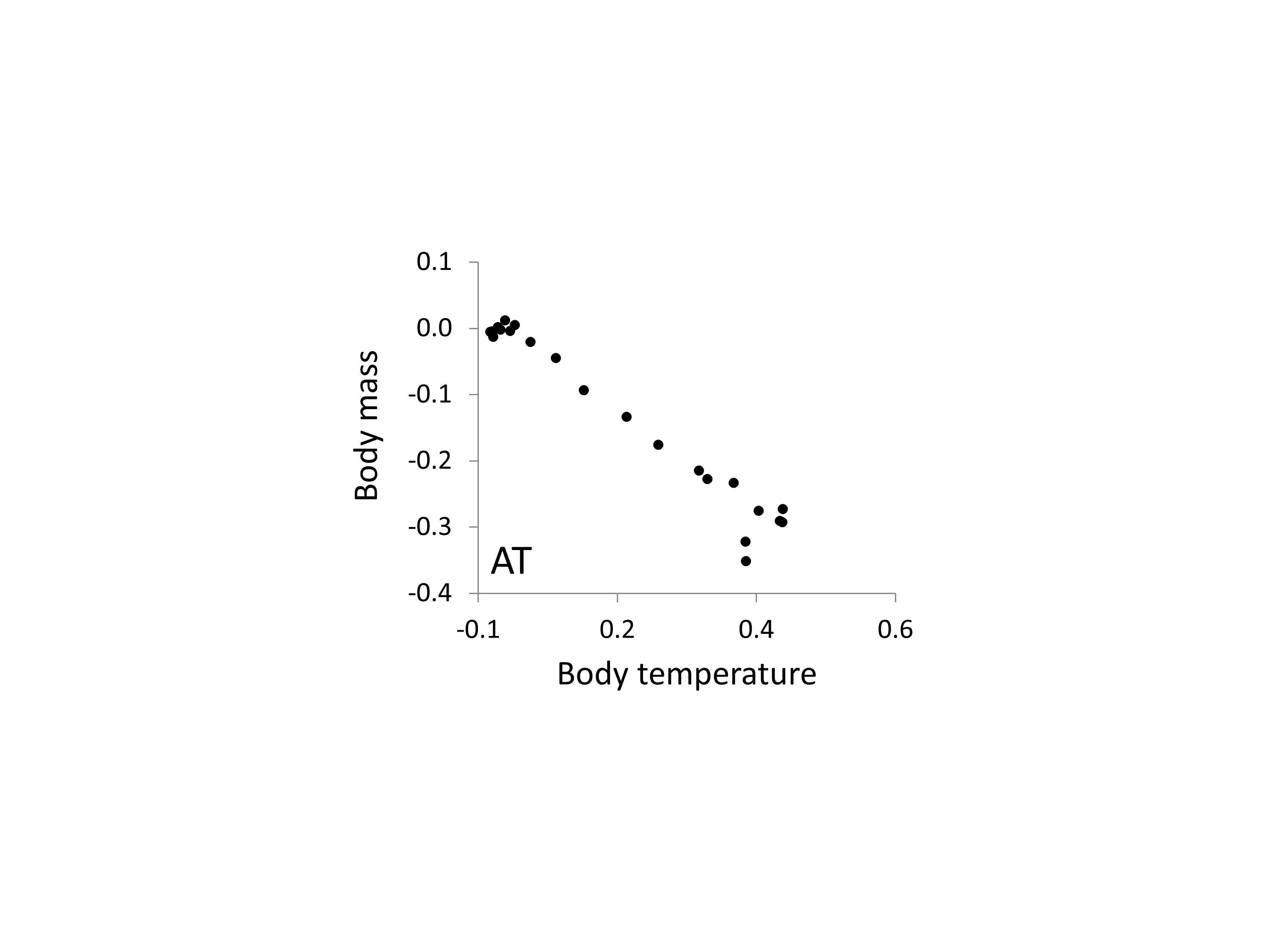

Supplement: Figure S8 — The two axes correspond to the estimates of the two phenotypes taken from Figs. 5 and 6 and show a strongly inverse relationship. [file peerj-02-663-s011.png]
